# Investigations on factors influencing HPO-based semantic similarity calculation

**DOI:** 10.1186/s13326-017-0144-y

**Published:** 2017-09-20

**Authors:** Jiajie Peng, Qianqian Li, Xuequn Shang

**Affiliations:** Northwestern Polytechnical University, 127 West Youyi Road, Xi’an, 710072 China

**Keywords:** Biological ontology, Semantic similarity, Human phenotype ontology

## Abstract

**Background:**

Although disease diagnosis has greatly benefited from next generation sequencing technologies, it is still difficult to make the right diagnosis purely based on sequencing technologies for many diseases with complex phenotypes and high genetic heterogeneity. Recently, calculating Human Phenotype Ontology (HPO)-based phenotype semantic similarity has contributed a lot for completing disease diagnosis. However, factors which affect the accuracy of HPO-based semantic similarity have not been evaluated systematically.

**Results:**

In this study, we proposed a new framework called *HPOFactor* to evaluate these factors. Our model includes four components: (1) the size of annotation set, (2) the evidence code of annotations, (3) the quality of annotations and (4) the coverage of annotations respectively.

**Conclusions:**

*HPOFactor* analyzes the four factors systematically based on two kinds of experiments: causative gene prediction and disease prediction. Furthermore, semantic similarity measurement could be designed based on the characteristic of these factors.

## Introduction

In the last few years, disease diagnosis has greatly benefited from the rapid development of next generation sequencing (NGS) technologies [1–3]. However, it is difficult to make the right diagnosis purely based on sequencing technologies for many diseases with complex phenotypes and high genetic heterogeneity. Because the genetic variants always relate to the complex clinical phenotypic characteristics. This kind of relation is difficult to understand [4–6].

Recently, tools to measure phenotypic characteristics have received increasing attention. Patient phenotypes are defined as the entire physical, biochemical and physiological makeup of a patient which determined by both genetically and environmentally [7]. Phenotype data can help people to understand the relation between the genetic variances and biological process activities. Advanced phenotype data analysis have played an important role in explaining gene function and understanding biological mechanism in biomedical research [8–11]. One of the key steps in phenotype data analysis is to precisely measure the similarity between phenotypes, and combine this knowledge with the disease diagnosis process to improve disease diagnosis efficiency. Therefore, a formal and controlled vocabulary is required to unify the representation of phenotypes and phenotype attributes.

It has been proved in many applications that ontology is effective to represent biomedical information as terms and their directed relationships with a directed acyclic graph (DAG) [12–18]. In order to meet the demand, an ontology called Human Phenotype Ontology (HPO) was constructed to describe the abnormal human phenotypes encountered in human Mendelian disease by Robinson et al. in 2008 [7]. Currently, HPO has been widely used to provide the unified and structured vocabulary to represent the phenotypic features encountered in human diseases [19]. HPO is always combined with next generation sequencing data analysis to support the human disease diagnosis [20, 21].

In order to improve diagnostic efficiency, many computational methods have been proposed to measure the phenotypic similarity between patient and historical disease data (or genes) [22, 23]. Among these computational measurements, calculating HPO-based phenotype semantic similarity has played an important role in completing disease diagnosis process.

Recently, several measurements have been developed to compute HPO-based phenotype semantic similarity [23–25]. Although ontology-based semantic similarity measurement has been extensively studied in the last ten years [26–33], it is still a difficult task to measure the phenotype similarity based on HPO structure and annotations. The reason is that many factors could affect the accuracy of HPO-based phenotype semantic similarity, such as the number of annotations per gene/disease, the evidence code of annotations, the coverage of annotations and the quality of annotations [22].

To figure out how different factors affect the performance of ontology-based semantic similarity measurement, some methods have been proposed to evaluate different involved factors. To test whether different editions of Gene Ontology (GO) would result in different semantic similarities, Gillis et al. proposed an evaluation framework based on protein interaction networks [34]. The result shows that 3 and 20% of genes are not semantically similar to themselves between monthly GO editions and between biennially GO editions. The semantic similarities are only stable over short-period GO editions. Skunca et al. proposed a novel method to systematically evaluate the quality of the computationally inferred GO annotations [35]. The reliability of electronic GO annotations is defined as the proportion of electronic annotations confirmed by the experimental annotations in the future release of GO. The coverage is defined as the proportion of experimental annotations predicted by the electronic annotations in an older release of GO. The result shows that the electronic GO annotations have high quality, which could lead to accurate semantic similarity. Both of the aforementioned methods are based on the historical versions of ontology. These methods cannot be used to evaluate the factors that affect the performance of HPO-based semantic similarity measurement, since the historical versions of HPO are not available currently (personal communication with the founder of HPO). Furthermore, other factors may also affect the accuracy of HPO-based semantic similarity. First, HPO contains large numbers of annotations with different evidence code indicating the different levels of evidences supporting the annotation. Second, HPO is a growing data source. The coverage and quality of annotations may vary with the updating of HPO data source. Third, the number of HPO terms annotating different diseases/genes may be different. These factors are all related to the HPO-based semantic similarity calculation. It is difficult to evaluate each factor individually. It is challenging and demanding to test whether these factors would affect the accuracy of HPO-based semantic similarity. The evaluation of different factors may guide the design of HPO-based semantic similarity measurement and support the human disease diagnosis. However, to the best of our knowledge, no method has been proposed to evaluate the factors that affect the accuracy of HPO-based semantic similarity.

In this article, we proposed a new framework named *HPOFactor* to evaluate the effect of four factors involved in the HPO-based semantic similarity calculation separately. The contribution of our present study are as follows.

- To the best of our knowledge, *HPOFactor* is the first framework that is specially designed for evaluating the factors involved in HPO-based semantic similarity calculation;
- We develop a method to generate different versions of the HPO annotations with different coverage and quality levels;
- We test the minimal size of annotation set that does not affect the accuracy of HPO-based semantic similarity.

## Methods

**Fig. 1:**
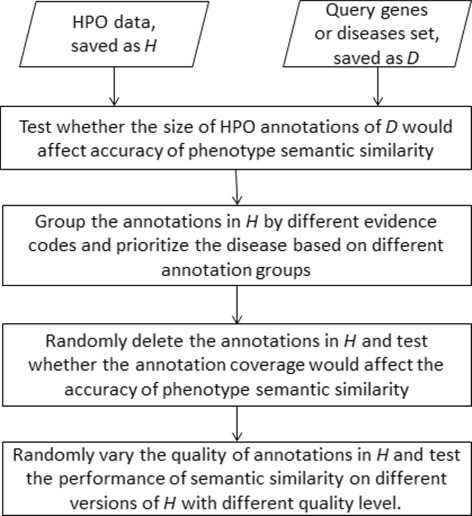
-The workflow of HPOFactor

We proposed *HPOFactor*, a new framework to evaluate the factors that affect the performance of phenotype semantic similarity measurement based on human phenotype ontology (HPO). The proposed framework has four parts. First, it tested whether changing the size of phenotype annotations would affect the performance of phenotype semantic similarity measurement. Second, it tested whether using annotations with different evidence codes would affect the performance of phenotype semantic similarity measurement. Third, it tested whether changing the annotation coverage would affect the performance of phenotype semantic similarity measurement by randomly deleting the HPO annotations. Last, it tested whether varying the quality of HPO annotations would affect the performance of phenotype semantic similarity measurement by randomly swapping the existing annotations of different HPO terms. The diagram of the whole framework is shown in Fig. 1.

### Calculating HPO-based semantic similarity

HPO provides a structured and controlled vocabulary to describe the human phenotypes and the genes/diseases associated with the phenotypes [7]. Using the unified description from HPO, the semantic similarity between gene and patient or between disease and patient can be calculated. Based on the HPO-based semantic similarity, we can predict whether a patient associates with a gene or has certain disease. For example, we can rank the candidate genes based on its similarity with the patient to predict the patient-associated genes. The phenotypes of a patient can be observed in clinical treatment and the gene/disease phenotype set can be obtained from database like HPO. Since the phenotype sets of patient, gene and disease are all able to be unified by HPO terms, calculating the similarity between patient and gene/disease is equal to calculating the similarity between two sets of HPO terms.

Let *P*_1_ and *P*_2_ be two phenotype term sets corresponding to a patient and a disease (or gene) respectively. *P*_1_ represents a set of phenotype terms of a patient observed in clinical treatment. *P*_2_ represents a set of phenotype terms of a disease (or gene) obtained from HPO database. Adopting the approach in [22], the semantic similarity between a patient and a gene (or disease) can be calculated by aggregating the pair-wise phenotype similarity between terms across *P*_1_ and *P*_2_. Given two phenotype sets, their HPO-based similarity is calculated as follows.

1 $$ {\begin{aligned} sim\left(P_{1},P_{2}\right)=&\frac{1}{2}\times sim_{set}\left(P_{1}\rightarrow P_{2}\right)+\frac{1}{2}\\&\times sim_{set}\left(P_{2}\rightarrow P_{1}\right) \end{aligned}}  $$

where *s* *i* *m*_*set*_(*P*_1_→*P*_2_) represents the similarity from *P*_1_ to *P*_2_. For each phenotype *p*_1_ in *P*_1_, we calculate the similarity between *p*_1_ and each phenotype in *P*_2_. Then the highest similarity score is selected as the similarity between *p*_1_ and phenotype set *P*_2_. The average of all similarities between each phenotype in *P*_1_ and *P*_2_ is defined as the similarity from *P*_1_ to *P*_2_. Mathematically, *s* *i* *m*_*set*_(*P*_1_→*P*_2_) is defined as follows.

2 $$ {{} \begin{aligned} sim_{set}(P_{1}\rightarrow {P_{2}})\,=\,avg \left[ \sum_{p_{1}\in P_{1}} max_{p_{2}\in P_{2}} sim_{term}(p_{1},p_{2})\right] \end{aligned}}  $$

where *s* *i* *m*_*term*_(*p*_1_,*p*_2_) represents semantic similarity between two phenotypes *p*_1_ and *p*_2_. It is noted that the similarity from phenotype set *P*_1_ to *P*_2_ is different from the similarity from phenotype set *P*_2_ to *P*_1_. Therefore, Eq. 1 averages the two dissymmetric similarities as the similarity between two phenotype sets.

To calculate *s* *i* *m*_*term*_(*p*_1_,*p*_2_), let *S*(*p*_1_,*p*_2_) be the set of all common ancestors of *p*_1_ and *p*_2_. *p*_*min*_ is the term that has the minimal annotations in *S*(*p*_1_,*p*_2_). Given two phenotypes *p*_1_ and *p*_2_, their similarity *s* *i* *m*_*term*_(*p*_1_,*p*_2_) is defined as follows.

3 $$\begin{array}{@{}rcl@{}} sim_{term}(p_{1},p_{2}) = -\log\frac{N_{p_{min}}}{N} \end{array} $$

where $N_{p_{min}}$ is the number of annotations of *p*_*min*_ (including annotations of itself and its descendants) and *N* is the total number of annotations involved in HPO.

Based on this semantic similarity measurement, we will evaluate the factors that affect HPO-based semantic similarity in the following subsections.

### Test the effect of the size of annotation set

In the process of calculating semantic similarity measurement introduced in last subsection, one of the key factors is the size of annotation set of compared genes or diseases. The size is usually large in the HPO branches for those well studied ones. Therefore, the size of annotation set is not a stable factor in the semantic similarity calculation. In this subsection, we proposed a method to test whether the size of annotation set would affect the precision of semantic similarity.

Given a set of query patients *Q*, each element *q* in *Q* has an annotation set obtained from clinical treatment saved as *P*_*q*_. Given a set of genes/diseases *H* involved in HPO database, each element *h* in *H* has an annotation set obtained from HPO database saved as *P*_*h*_. We changed the size threshold of annotations *s* and calculate the semantic similarity at different thresholds. Given the threshold *s*, the detail of the method is described as follows. For each element *h* in *H*, we randomly selected *s* phenotypes from *P*_*h*_, saved as $P^{s}_{h}$. This step is represented mathematically in Eq. 4.

4 $$ P^{s}_{h} = RandomSelection(P_{h},s)  $$

For each query patient *q* in *Q*, we calculate the similarity between *P*_*q*_ and $P^{s}_{h}$ for each *h* in *H* using Eq. 1. Then, we ranked all elements in *H* based on the similarities with *P*_*q*_ saved as *H*_*order*_ (see Eq. 5).

5 $$ H_{order} = Rank\left(H, \{y|y=sim(P_{q},P^{s}_{h}),h \in H \}\right)  $$

At last, we can test whether the known patient associated element (gene or disease) has a high rank in *H*_*order*_. The higher the rank is, the better the performance of the semantic similarity measurement is.

### Test the effect of annotations with different evidence codes

In HPO, the annotations are supported by different evidences. When the HPO project was initialed, most annotations were extracted from the OMIM database [36] by parsing the clinical features. These annotations are labeled by the evidence code IEA representing “inferred from electronic annotation”. There are also other evidences, such as PCS representing “inferred from public clinical study and biomedical literature”, ICE representing “inferred from individual clinical experience”, ITM representing “inferred by text-mining technique” and TAS representing “inferred from traceable author statement”.

In this subsection, we test whether using different annotations with different evidence codes would affect the precision of HPO-based semantic similarity. First, the annotations in HPO are grouped based on the evidence codes. Given the annotation set *A*, *A*_*e*_ represents the annotation set with evidence *e*. For each evidence code *e*, we only use annotations contained in *A*_*e*_ to calculate the semantic similarity between phenotypes. Given a set of genes/diseases *H*, the annotation set of each element *h* in *H* is obtained from *A*_*e*_, saved as *P*_*he*_. Similar with the process described in last subsection, we rank all elements in *H* based on the similarities with the phenotypes of query patient.

6 $$ H_{order} = Rank\left(H, \{y|y=sim(P_{q},P_{he}),h \in H \}\right)  $$

Finally, we could see which evidence code can lead the best performance.

### Test the effect of annotation quality

To determine whether annotation quality was one of the factors that control the performance of HPO-based semantic similarity, we re-ran semantic similarity measurement by varying the quality of HPO annotation. To this end, we varied the HPO annotation quality by randomly swapping the phenotype-annotation associations in HPO. For example, assume that *d*_1_→*p*_1_ and *d*_2_→*p*_2_ are two disease-phenotype pairs randomly selected from HPO. After the swapping process, we get two new pairs *d*_1_→*p*_2_ and *d*_2_→*p*_1_ to replace the original two pairs. Given the original HPO annotation set *A*, we can generate a low quality set *A*_*u*_ by randomly swapping the phenotype-annotation associations. To make sure the quality be decreased, the new generated phenotype-annotation associations should not be contained in the set of original HPO phenotype-annotation associations. *u* represents different quality levels, such as swapping 20% phenotype-annotation associations, 40% phenotype-annotation associations. *A*_*u*_ has the same size with *A* but different quality level. For each low quality level *u*, we use the low quality annotation set *A*_*u*_ to calculate the semantic similarity between phenotypes. The annotation set of each element *h* in *H* is got from *A*_*u*_, saved as *P*_*hu*_. Comparison of the performance of semantic similarity using annotation sets with different quality level could test whether the annotation quality was a key factor of the HPO-based semantic similarity measurement.

### Test the effect of annotation coverage

Currently, HPO is not complete. Much unknown knowledge and knowledge in the literature are not included in the HPO database. Therefore, it is critical to test whether annotation coverage was a key factor for HPO-based semantic similarity measurement. To this end, we randomly delete the annotations from annotation set *A* to generate a low coverage annotation set *A*_*c*_. *c* represents different coverage levels, such as randomly deleting 20% of the annotations in *A*, deleting 40% of the annotations in *A*. For each coverage level *c*, we use the low coverage annotation set *A*_*c*_ to calculate the semantic similarity between phenotypes. Given a set of genes/diseases *H*, the annotation set of each element *h* in *H* is obtained from *A*_*c*_, saved as *P*_*hc*_. By comparing the results on the annotation sets with different coverage levels, we can test whether the annotation coverage is a key factor for HPO-based semantic similarity calculation.

## Results

### Data preparation

The Human Phenotype Ontology (HPO) data used in our experiment was downloaded from the HPO official website (http://human-phenotype-ontology.github.io/) on April 1st, 2016. It includes 459,452 gene annotations and 78,313 disease annotations. *HPOFactor* was implemented with Python language.

We used the curated clinical phenotype features in [22] to generate simulated patients for experiments. The associated phenotypes, disease causative genes and penetrance of each phenotype of the diseases are available in the dataset. For each disease, we simulated 100 patients. The simulation process is described as follows. To consider the gender-specificity of phenotypes, we first simulated the gender of each patient. A random number *f*_*g*_ was generated. Then, the patient’s gender is assigned as follows:

7 $$ \left\{\begin{array}{ll} f_{g}>0.5 & \text{,male} \\ f_{g}\leq 0.5 & \text{,famale} \end{array}\right.  $$

Second, given a phenotype *p* of a patient, a random number *r*_*p*_ was generated. Let *f*_*p*_ be the penetrance of this phenotype associated with the assigned disease. If *r*_*p*_<*f*_*p*_, the phenotype *p* was assigned to the patient. It is noted that each simulated patient must have at least one phenotype. Finally, 3300 patients was generated. For each patient, we know its disease causative gene and associated disease. Therefore, we adopted the evaluation criterion from [22] to test whether the causative gene or associated disease of a patient can be identified based on the HPO-based semantic similarity.

### Evaluation for the size of annotation set

**Fig. 2:**
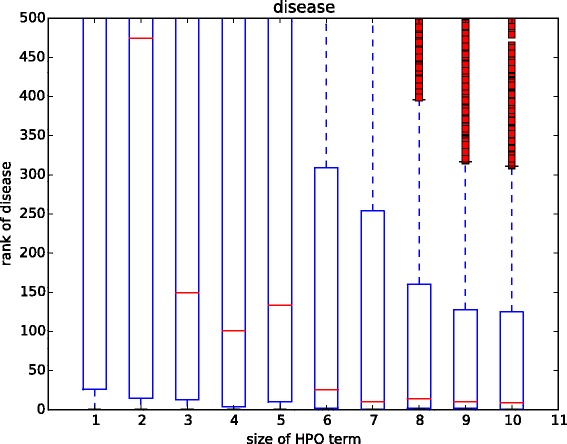
-The rank of disease by changing the size of phenotype annotation set. The x-axis is the number of HPO annotations. The y-axis is the rank of disease associated with the query patient

In this experiment, we compared the results of using different sizes of annotation set to identify the disease associated with the patient. The size threshold *s* used in Eq. 4 is from 1 to 10. The result shows that the patient associated diseases have low ranks when the number of annotations is small, indicating low performance (see Fig. 2). Particularly, when *s*=1 and *s*=2, the ranks of most true patient associated diseases are lower than the 450. Figure 2 shows that the performance improved with the increase of the size of annotation set. Noted that the performance become stable when *s*>5.

**Fig. 3:**
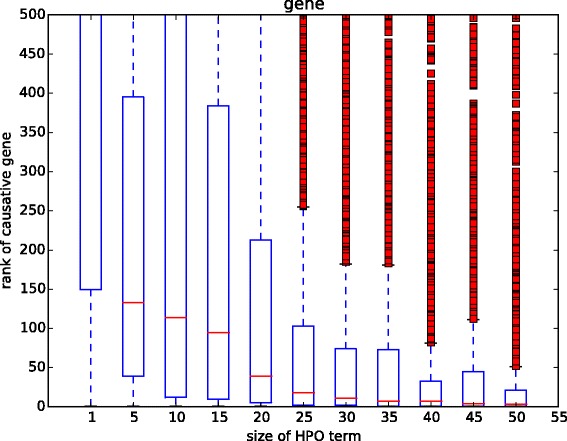
-The rank of causative gene by changing the size of phenotype annotation set. The x-axis is the number of HPO annotations. The y-axis is the rank of causative gene of the query patient

We also compared the results of using different sizes of annotation set to identify the causative gene. The gene annotations in HPO are richer than the disease annotations (see the Data preparation subsection). To see the global distribution, we set the gene set threshold *s* as {1,5,10,…,45,50}. Similar with the result of identifying disease, the causative genes have low ranks when the number of annotations is small (see Fig. 3). When *s*=1, the ranks of most causative genes are lower than 500. It is shown that the performance of HPO-based semantic similarity improved steadily with the increase of the number of annotations. The performance keeps stable when the size of annotations is larger than 25.

The result shows an important guidance for the HPO-based semantic similarity calculation that the result may be more reliable when the number of annotations is large enough.

### Evaluation for the annotations with different evidence codes

In this part, we test whether using annotations with different evidence codes would affect result of identifying the disease associated with the patient. We do not test the performance for causative gene identification since the gene annotations in HPO do not have evidence codes currently. We only compare three evidence codes: IEA, TAS and PCS, since other evidence codes do not have enough number of annotations. To avoid the bias resulting from the lack of annotation, we did the experiment on the size of annotations sets which are larger than 5. We choose the size threshold since the experiment in last subsection shows that the performance become stable when *s*>5.

**Fig. 4:**
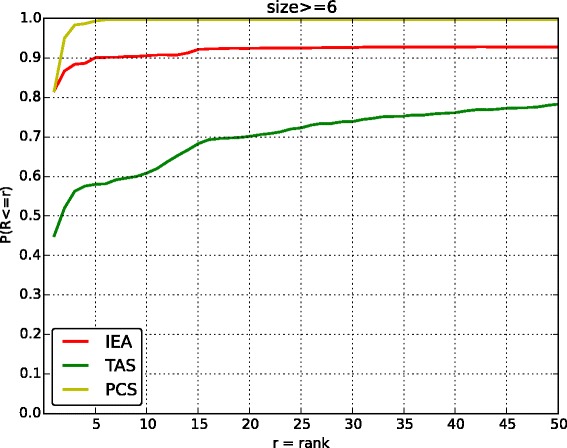
-The rank of disease by the phenotype with different evidence code. The x-axis is the ranking threshold for the disease. The y-axis is the ratio of patients satisfying the ranking threshold

Figure 4 shows that using annotations with *PCS* evidence code performs better than using the annotations with *IEA* and *TAS* evidence code. Specifically, when the ranking threshold is 5, the ratio of patients for *PCS* is 0.993, which is higher than *IEA* and *TAS* (the number is 0.901 and 0.580 respectively). The ratio of patients for *PCS* is 0.997, when the ranking threshold is 10. In comparison, the ratios of patients satisfying the threshold are 0.906 and 0.609 for *IEA* and *TAS* respectively.

### Evaluation for the annotation quality

**Fig. 5:**
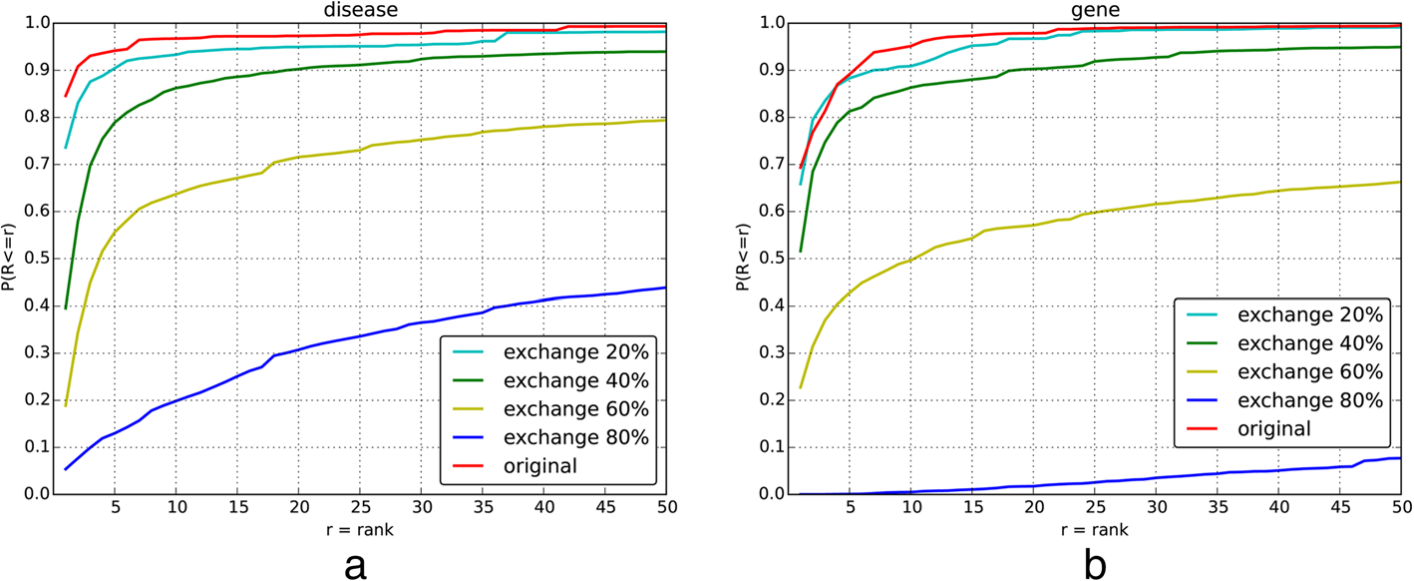
-The rank of disease (**a**) and causative gene (**b**) by varying the quality of phenotype annotations. The x-axis is the ranking threshold for the disease/causative gene. The y-axis is the ratio of patients satisfying the ranking threshold

To test the effect of annotation quality to the performance of HPO-based semantic similarity, we compared the results of using annotation sets with different qualities to identify the patient associated diseases (Fig. 5(a)) or causative genes (Fig. 5(b)). Overall, the result shows that the performance goes down with the decrease of the annotation quality in both experiments. It is shows that the performance decreases significantly when more than 40% annotations become noise.

In the associated disease identification experiment, when the ranking threshold is 10, the ratio of patients satisfying the threshold is 0.967 for original annotation set. In comparison, the ratios of patients satisfying the threshold are 0.933, 0.862, 0.637 and 0.198 for annotation sets with 20%, 40%, 60% and 80% noise respectively. Furthermore, the statistical test shows that the result for original annotation set is significantly different with 40%, 60% and 80% set (Tukey test, *p*-value <0.05).

In the causative gene identification experiment, when the ranking threshold is 10, the ratio of patients satisfying the threshold is 0.951 for original annotation set. In comparison, the ratios of patients satisfying the threshold are 0.909, 0.863, 0.496 and 0.005 for annotation sets with 20%, 40%, 60% and 80% noise respectively. Furthermore, the statistical test shows that the result for original annotation set is significantly different with 40%, 60% and 80% set (Tukey test, *p*-value <0.05).

### Evaluation for the annotation coverage

**Fig. 6:**
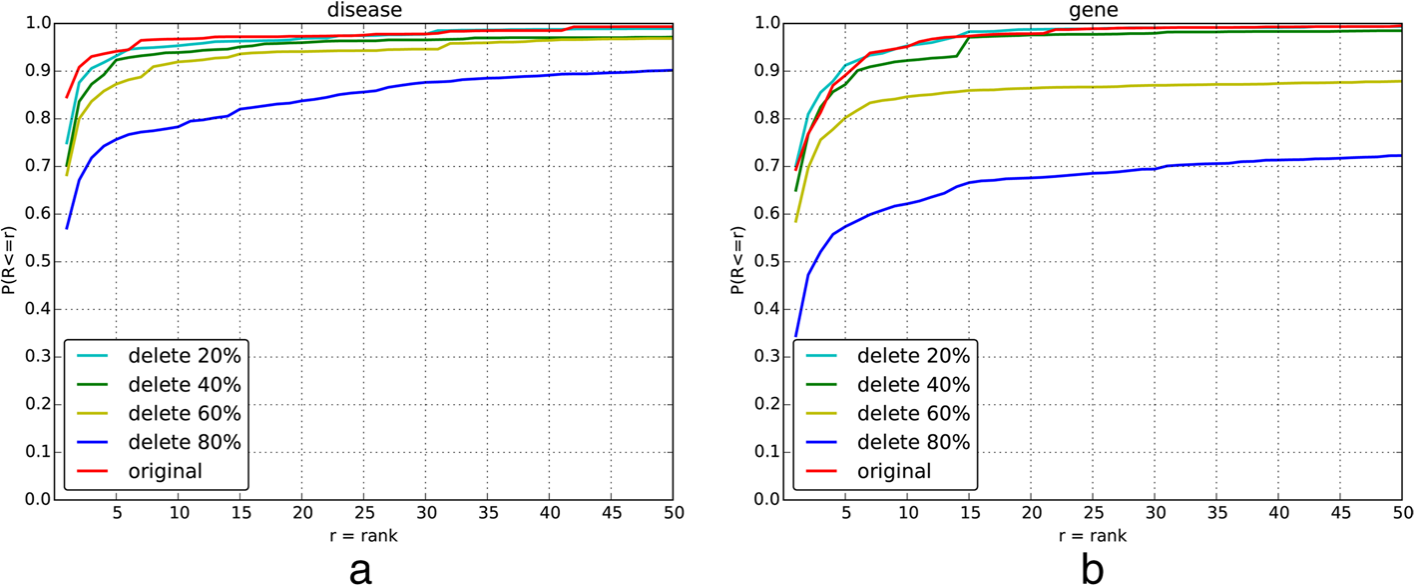
-The rank of disease (**a**) and causative gene (**b**) by changing the coverage of phenotype annotations. The x-axis is the ranking threshold for the disease/causative gene. The y-axis is the ratio of patients satisfying the ranking threshold

To test the effect of annotation coverage to the performance of HPO-based semantic similarity, we randomly delete the annotations and use annotation sets with different coverage levels to identify the associated disease and causative genes. The result shows that the performance of HPO-based semantic similarity decreased with the reduction of the annotations (Fig. 6(a) and (b)). However, there was no significant difference when the deleted annotations are less than 60% (Tukey test, *p*-value >0.05). It indicates that HPO-based semantic similarity is more sensitive to the quality of annotations than the coverage of annotations.

## Discussion

In this article, we proposed a novel framework called HPOFactor to evaluate the factors that may affect the accuracy of HPO-based semantic similarity. HPOFactor evaluates four factors involved in the HPO-based semantic similarity: size of annotation set, evidence code of annotations, quality of annotations and coverage of annotations. Particularly, we found the performance of HPO-based semantic similarity decreased steadily with the reduction of coverage and quality of annotations. There was no significant difference among different coverage levels (*p*-value > 0.05), but there was significant difference among different quality levels (*p*-value < 0.05), indicating that quality is more important than coverage. This is important because not all human diseases and genes are annotated in current HPO, but existing annotations in HPO have high quality.

## Conclusion

Recently, the rapid development of next generation sequencing techniques have significantly accelerated disease diagnosis. However, it remains challenging to make the right diagnosis for many diseases with complex phenotypes and high genetic heterogeneity. Hence, HPO-based phenotype similarity become an important part of completing disease diagnosis.

The evaluation result can make the HPO-based semantic similarity better used in phenotype-based causative gene prediction and disease prediction. In the future, we will evaluate the combination effects of different factors on HPO-based semantic similarity. Furthermore, we will design semantic similarity measurement based on the characteristic of these factors.
